# Outcomes for Black and White Patients After Certification of Nearby Stroke Centers

**DOI:** 10.1001/jamanetworkopen.2025.22019

**Published:** 2025-07-28

**Authors:** Yu-Chu Shen, Anthony S. Kim, Renee Y. Hsia

**Affiliations:** 1 Department of Defense Management, Naval Postgraduate School, Monterey, California; 2 National Bureau of Economic Research, Cambridge, Massachusetts; 3 UCSF Weill Institute of Neurosciences, Department of Neurology, University of California, San Francisco; 4 Department of Emergency Medicine, University of California, San Francisco; 5 Philip R. Lee Institute for Health Policy Studies, University of California, San Francisco

## Abstract

**Importance:**

It is unclear whether stroke center certification near a community is associated with similar benefits for Black and White patients.

**Objective:**

To assess changes in admission to stroke centers, receipt of acute stroke treatment, and health outcomes for Black compared with White patients after the certification of a new stroke center near their community.

**Design, Setting, and Participants:**

This cohort study comprises Medicare fee-for-service patients from urban US communities with a primary diagnosis of acute ischemic stroke between January 1, 2009, and December 31, 2019, and compares changes in outcomes. Statistical analysis was performed from September 2024 to April 2025.

**Exposure:**

When a patient’s community was exposed to the certification of a nearby stroke center relative to a patient of the same self-reported race in a community that was not exposed to a stroke center certification.

**Main Outcomes and Measures:**

A difference-in-differences framework was used to compare changes in (1) stroke center admission, (2) receipt of acute stroke treatments (thrombolytics and thrombectomy), and (3) health outcomes (home at 90 days and 1-year mortality) among Black and White patients. The empirical model was a linear probability model with community fixed effects.

**Results:**

This study included 2.1 million patients with stroke (15% Black patients and 85% White patients; 57% women; 15% aged 65-69 years, 16% aged 70-74 years, 18% aged 75-79 years, 19% aged 80-84 years, and 32% aged ≥85 years). After a newly certified stroke center opened near a community, the probability of admission to the stroke center increased for both White patients (15.6 percentage points [pp]; 95% CI, 14.6-16.6 pp) and Black patients (14.6 pp; 95% CI, 13.2-15.9 pp). Thrombolytic therapy increased for White patients by 0.2 pp (95% CI, 0.0-0.4 pp) but decreased for Black patients by 0.4 pp (95% CI, −0.7 to −0.1 pp) compared with their control communities. Black patients also had a 0.5-pp decrease in thrombectomy (95% CI, −0.6 to −0.3 pp). One-year mortality remained stable for White patients, while mortality for Black patients increased by 0.5 pp (95% CI, 0.1-0.9 pp).

**Conclusions and Relevance:**

This cohort study of 2.1 million patients with stroke found that stroke center certification near a community was associated with similar increases in rates of admission to the stroke center for Black and White patients, but it was associated with increased rates of receiving thrombolytics only for White patients. Thrombolysis and thrombectomy rates did not increase for Black patients who were exposed to a newly certified stroke center nearby. This study suggests that, despite the expansion of certified stroke centers, disparities in stroke care between Black and White patients persist.

## Key Points

**Question:**

Is stroke center certification near a community associated with the same benefits in access to care, receipt of treatments, and health outcomes for Black and White patients?

**Findings:**

In this cohort study including 2.1 million patients with stroke, certification of nearby stroke centers was associated with an increase in the likelihood of admission to the stroke centers for both Black and White patients, but it was associated with increased rates of receiving thrombolytic therapy only for White patients. Black patients had lower rates of both thrombolytic therapy and thrombectomy, along with higher 1-year mortality, compared with Black patients in reference communities.

**Meaning:**

This study suggests that although the certification of nearby stroke centers was associated with an increase in admissions to the stroke centers for Black and White patients, it was associated with increased receipt of acute stroke treatment only among White patients, highlighting persistent inequities.

## Teaser

This cohort study assess patterns in admission to stroke centers, receipt of acute stroke treatment, and health outcomes for Black compared with White Medicare fee-for-service patients after the certification of a new stroke center near their community.

## Introduction

Over the past few decades, marked advances have transformed stroke care, turning what was once considered an untreatable condition into one for which therapies can significantly improve outcomes. The introduction of intravenous thrombolytics in the 1990s^1^ and endovascular thrombectomy in 2015 marked key milestones in reducing disability and improving functional recovery among eligible patients with stroke.^2^ However, these interventions require substantial resources, including imaging technology, specialized medical devices, and trained personnel. As a result, advanced stroke care is available only at certain hospitals, often designated by state or national certification. These certifications range from Acute Stroke Ready Hospitals (ASRHs), which provide initial diagnosis and stabilization, to Comprehensive Stroke Centers (CSCs), which can perform advanced interventions, such as thrombectomy.

Access to certified stroke centers is not distributed equitably.^3^ Nearly half of all hospitals in the US lack stroke certification, and prior studies show that higher-level stroke centers are more often located in wealthier areas,^3,4,5^ while Black and racially and ethnically segregated communities have lower per-capita access.^5,6,7^ These inequities are associated with disparities in treatment and outcomes among historically underserved groups, including Black, Hispanic, and socioeconomically disadvantaged patients.^8^

What remains unknown is whether the certification of a nearby stroke center is associated with equal benefits for Black and White patients. This question is critical for informing equitable policy decisions around resource allocation. To address this, we analyzed 11 years of patient-level data from Medicare (2009-2019) to assess patterns in admission, treatment, and outcomes by race after stroke center certification.

## Methods

### Data Sources

This retrospective, observational cohort study was approved by the National Bureau of Economic Research institutional review board and was deemed exempt from patient consent under exemption category 4 as detailed at 45 CFR46.104(d)(4). This study followed the Strengthening the Reporting of Observational Studies in Epidemiology (STROBE) reporting guideline. We combined patient-, hospital-, and community-level data for our analysis. Patient-level data were obtained from national 100% Medicare Provider and Analysis Review files and Medicare Beneficiary Summary Files from January 1, 2009, through December 31, 2019, which contain the following relevant patient information: admission date, diagnoses, procedure codes, procedure dates, sex, self-reported race and ethnicity,^9^ age at time of admission, mailing zip code (proxy for community), and date of death (if applicable). We merged these data with US Census and American Community Survey data to define zip code–level community characteristics and identify geographic coordinates. Patients from rural communities were excluded using Federal Office of Rural Health Policy^10^ designations. Hospital stroke certification status and dates were obtained from national and state accrediting bodies.^11^ Finally, we derived a drive-time database using web-based queries under normal traffic conditions from the geographic center of each patient’s zip code to stroke centers based on each location’s geographic coordinates (longitude and latitude).^12^

### Study Population

Our patient population included all Medicare fee-for-service patients whose primary discharge diagnosis was acute ischemic stroke and who were admitted to hospitals between January 1, 2009, and December 31, 2019. Patients were identified using the following *International Classification of Diseases, Ninth Revision* codes 433.x1, 434.x1, or 436 and *International Statistical Classification of Diseases and Related Health Problems, Tenth Revision* code I63, as done previously.^13,14,15^ To properly estimate the key associations, we applied several exclusion criteria. First, we excluded patients whose mailing zip code was more than 160 km (100 miles) from their hospital admission, which likely indicated treatment while away from home or inaccurate residential data. Second, we limited our analysis to patients residing in urban communities, where stroke care expansion differs from rural areas.^16^ Third, we restricted our sample to those who self-identified as Black or White, the focus of our disparity analysis. Fourth, we excluded patients enrolled in Medicare due to end-stage kidney disease, those younger than 65 years, and those without continuous Medicare Part A coverage within the past 12 months, to reduce selection bias. Fifth, we limited our analysis to patients residing in communities with at least 100 patients across the study period to ensure an adequate sample size within each community.

### Determining a Hospital’s Stroke Center Certification Status and a Community’s Exposure to Newly Certified Stroke Centers

We determined if and when a hospital became certified as a stroke center and at what level using data from 4 Centers for Medicare & Medicaid Services–approved accrediting organizations: The Joint Commission, Det Norske Veritas, the Accreditation Commission for Health Care (formerly the Healthcare Facilities Certification Program), and the Center for Improvement in Healthcare Quality, as well as state departments of health.^11^ Following prior work, we categorized hospitals by certification level (from least to most advanced stroke care capacity): not certified, ASRH, Primary Stroke Center (PSC), Thrombectomy-Capable Stroke Center (TSC), or CSC.

We defined a community as being exposed to a newly certified stroke center on and after the quarter when a stroke center became newly certified within a 30-minute drive time to the community’s center (based on longitude and latitude) using the following steps. First, we used a drive-time database to identify all operating hospitals within a given community’s 30-minute drive-time range for each quarter. Second, we evaluated quarter-by-quarter changes in stroke center status for each set of hospitals and classified communities according to whether there was a newly certified stroke center and the highest level of stroke center certification in that quarter. We chose a 30-minute range given the time-sensitive nature of the effectiveness and risks associated with acute stroke interventions and prior work on travel times for medical treatments, including emergency cesarean delivery,^17^ cardiac care,^18,19^ and primary care.^20^ In our sensitivity analysis, we changed the threshold to 15 minutes to account for potentially closer proximity in urban communities.

### Outcomes

Our goal was to investigate whether Black and White patients had differential benefits associated with their community’s exposure to a newly certified stroke center nearby in terms of actual access to stroke centers (ie, admission to a stroke center), receipt of acute stroke treatment (specifically, receipt of intravenous thrombolytics and/or endovascular thrombectomy), and health outcomes (defined as home at 90 days [not in an inpatient acute or rehabilitation hospital] and 1-year mortality).

### Statistical Analysis

Statistical analysis was performed from September 2024 to April 2025. We used a difference-in-differences framework, which follows the same principle as a case crossover design. In essence, we compared changes in patient outcomes between the preperiod and the postperiod in the treatment community (those who were exposed to newly certified stroke centers within a 30-minute drive) with changes in outcomes among patients in the control community (no exposure). The advantage of following this difference-in-differences framework is that it compared changes in outcomes within each community over time, thereby accounting for baseline differences between treatment and control communities. It also accounted for secular trends that are common to both groups. To follow this framework, we implemented a linear probability model (LPM) with community fixed effects.

In model 1, the key independent variable was a set of binary indicators that took on the value 1 on and after the year-quarter a community was exposed to a newly certified stroke center (regardless of the certification level). We estimated this coefficient separately for Black and White patients. In model 2, we considered 3 stroke center certification level groupings: whether the highest level of a certified stroke center nearby was ASRH, PSC, or TSC or CSC, also separately for Black and White patients.

We intentionally chose the LPM over the logit models because the LPM can consistently estimate the association between changes in stroke center access and dichotomous outcomes in panel data when there is a large number of community fixed effects. In contrast, a logit model or other models that rely on maximum likelihood estimation (such as probit or modified Poisson models), while appropriate for cross-sectional data, would result in inconsistent estimates in a panel data setting due to the inclusion of a large number of fixed effects.^21^ The community fixed effects were critical to our identification strategy because they controlled for systematic unobserved differences across communities, including any baseline differences in underlying patient health and socioeconomic conditions. To address the concern that the LPM produces overly narrow 95% CIs, we estimated heteroskedasticity-robust SEs that are clustered at the community level (ie, it allows for correlation among patients from the same community).^22^ Across all models, we included time dummies to control for secular trends, patient demographic covariates (Black, female, age, and age squared), and 23 comorbid conditions, following prior work.^23,24^ We used Stata, version 18 (StataCorp LLC), for these analyses. All *P* values were from 2-sided tests and results were deemed statistically significant at *P* < .05.

## Results

Our final study population included 2 109 084 patients with stroke (15% Black patients and 85% White patients; 57% women and 43% men; 15% aged 65-69 years, 16% aged 70-74 years, 18% aged 75-79 years, 19% aged 80-84 years, and 32% aged ≥85 years) (Table). Fifty-six percent of White patients were female compared with 59% of Black patients. White patients with acute stroke were generally older than Black patients, with 13% of White patients aged 65 to 69 years compared with 25% of Black patients and 34% of White patients aged 85 years or older compared with only 19% of Black patients. Overall, 87% of White patients and 93% of Black patients lived in communities that gained a certified stroke center within a 30-minute drive time during the study period. Although 2% of patients in both groups lived near a hospital that became an ASRH, a higher share of White patients lived near newly certified PSCs (White patients, 20%; Black patients, 13%). Comorbidity profiles also differed by race. Black patients had higher rates of diabetes (Black patients, 45%; White patients, 30%), kidney failure (Black patients, 26%; White patients, 18%), and hypertension (Black patients, 91%; White patients, 84%). In contrast, White patients more often had hypothyroidism (White patients, 19%; Black patients, 8%) and depression (White patients, 10%; Black patients, 6%). We controlled for these factors in our models.

**Table.** Descriptive Statistics of Patient Characteristics

Characteristic	Patients, No. (%)	
	White (n = 1 785 789)	Black (n = 323 295)
Lives in a community where a hospital within a 30-min driving distance received stroke center certification during study period (2009-2019)		
Overall	1 557 739 (87)	301 062 (93)
By highest certification level		
ASRH	43 018 (2)	5417 (2)
PSC	354 483 (20)	40 759 (13)
TSC or CSC	1 160 238 (65)	254 886 (79)
Patient demographics		
Sex		
Female	1 005 147 (56)	191 971 (59)
Male	780 642 (44)	131 324 (41)
Age distribution at time of admission, y		
65-69	239 629 (13)	82 243 (25)
70-74	278 501 (16)	67 588 (21)
75-79	312 450 (17)	60 192 (19)
80-84	346 407 (19)	50 320 (16)
≥85	608 802 (34)	62 952 (19)
Patient clinical conditions		
Recurring stroke	148 525 (8)	36 856 (11)
Transfer from another hospital	91 435 (5)	11 961 (4)
Peripheral vascular disease	182 776 (10)	30 434 (9)
Pulmonary circulation disorders	63 264 (4)	12 472 (4)
Diabetes	528 898 (30)	144 846 (45)
Kidney failure	313 883 (18)	85 402 (26)
Liver	16 934 (1)	3971 (1)
Cancer	72 969 (4)	13 277 (4)
Dementia	190 040 (11)	39 653 (12)
Valvular disease	186 087 (10)	23 104 (7)
Hypertension	1 497 140 (84)	294 013 (91)
Chronic pulmonary disease	290 415 (16)	44 817 (14)
Rheumatoid arthritis or collagen vascular disease	52 033 (3)	7160 (2)
Coagulation deficiency	67 499 (4)	12 984 (4)
Obesity	139 563 (8)	34 552 (11)
Substance use	36 623 (2)	10 676 (3)
Depression	186 913 (10)	18 818 (6)
Psychosis	128 612 (7)	16 540 (5)
Hypothyroidism	344 380 (19)	27 017 (8)
Paralysis and other neurologic disorder	1 019 345 (57)	190 175 (59)
Ulcer	5847 (0.3)	1193 (0.4)
Weight loss	74 349 (4)	19 366 (6)
Fluid and electrolyte disorders	398 284 (22)	85 745 (27)
Anemia (blood loss and deficiency)	209 148 (12)	59 330 (18)

Abbreviations: ASRH, Acute Stroke Ready Hospital; CSC, Comprehensive Stroke Center; PSC, Primary Stroke Center; TSC, Thrombectomy-Capable Stroke Center.

**Figure 1.**
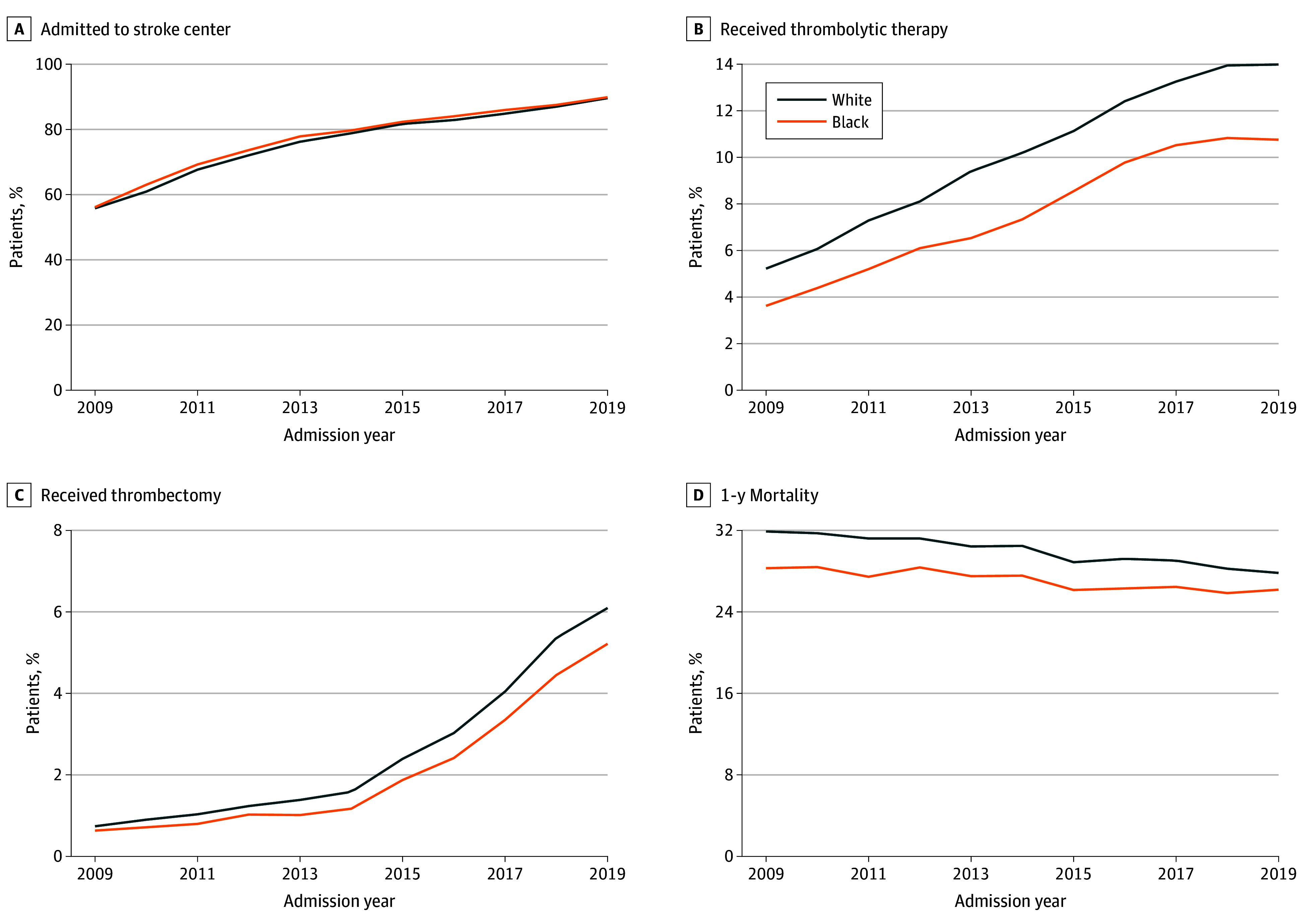
Time Trend in Outcomes Between Black and White Patients With Acute Stroke

Figure 1 shows the time trend in selected outcomes between Black and White patients. In Figure 1A, stroke center admission probabilities were similar between racial groups. Figure 1B shows that receipt of thrombolytic therapy increased over time but remained lower for Black patients; in 2009, 5% of White patients and 4% of Black patients received thrombolytics, but by 2019, 14% of White patients and 11% of Black patients received thrombolytics. Figure 1C shows that thrombectomy rates increased similarly for both groups, with thrombectomy rates increasing from 1% in 2009 to 6% in 2019 for White patients and increasing from 1% to 5% for Black patients. Due to a younger age profile, Black patients had a lower unadjusted 1-year mortality rate than White patients overall, but this gap decreased from 32% in 2009 to 28% in 2019 for White patients and from 28% in 2009 to 26% in 2019 for Black patients (Figure 1D).

**Figure 2.**
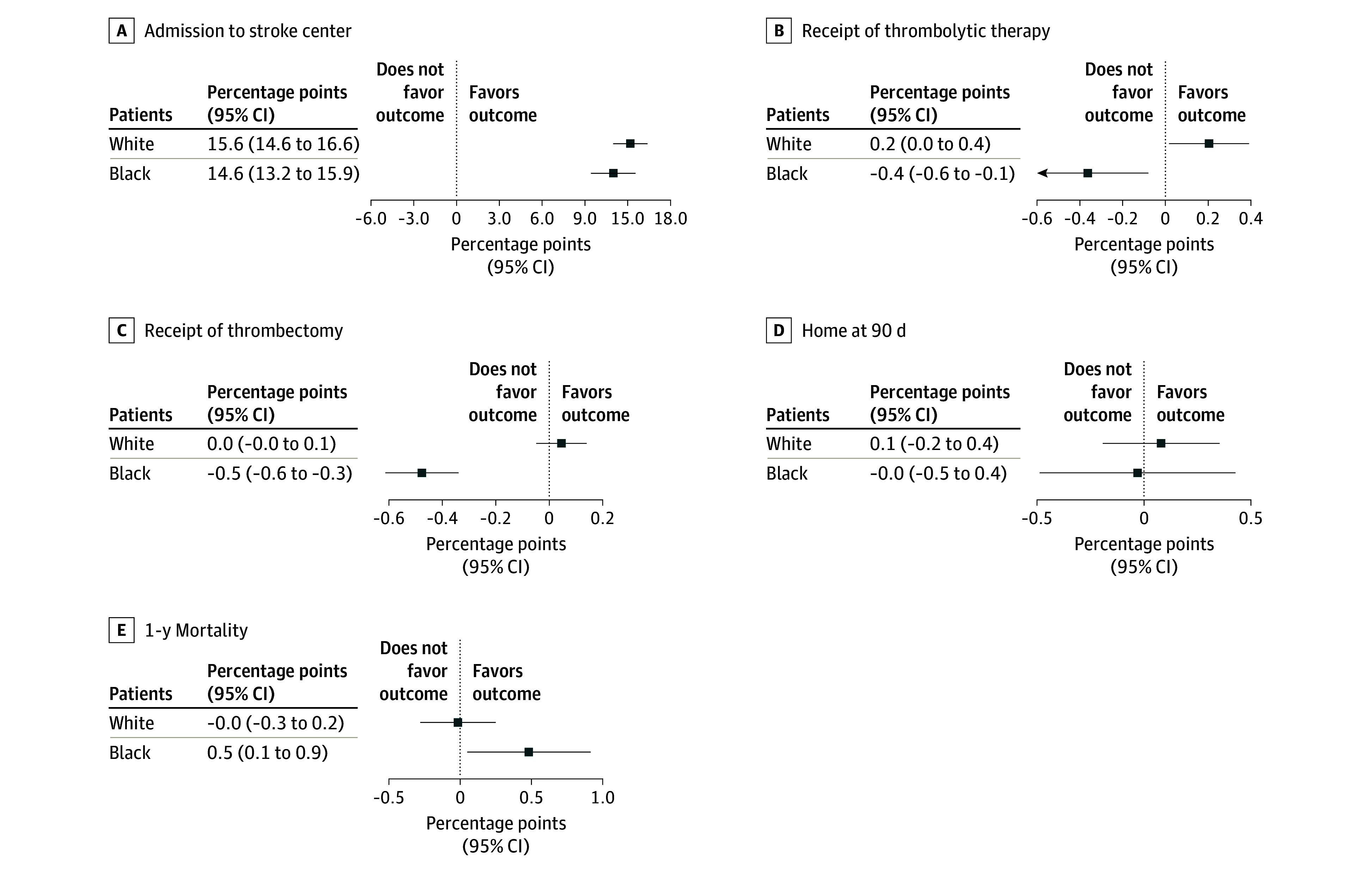
Changes in Probability of Outcomes After Patient Exposure to Newly Certified Stroke Center Within a 30-Minute Drive Time Error bars indicate 95% CIs.

Figure 2 shows the changes in outcomes for White and Black patients when their communities were exposed to newly certified stroke centers relative to White and Black patients in communities where there was no change in stroke center capacity nearby (full results in eTable 1 in Supplement 1). The percentage of patients admitted to a nearby stroke center increased similarly for both groups after certification: 15.6 percentage points (pp) (95% CI, 14.6-16.6 pp) for White patients and 14.6 pp (95% CI, 13.2-15.9 pp) for Black patients, from a 58% mean baseline (Figure 2). The probability of receiving thrombolytic therapy increased for White patients by 0.2 pp (95% CI, 0.0-0.4 pp) from a baseline of 5% (equivalent to a 4% relative increase) but decreased for Black patients by 0.4 pp (95% CI, −0.7 to −0.1 pp). The likelihood of receiving a thrombectomy did not change for White patients but decreased by 0.5 pp (95% CI, −0.6 to −0.3 pp) among Black patients relative to Black patients in the control community, from a baseline mean of 1%. We did not observe significant changes in rates of being home at 90 days or 1-year mortality for White patients, but we found that 1-year mortality among Black patients was slightly higher when their community was exposed to newly certified stroke centers (0.5 pp; 95% CI, 0.1-0.9 pp).

**Figure 3.**
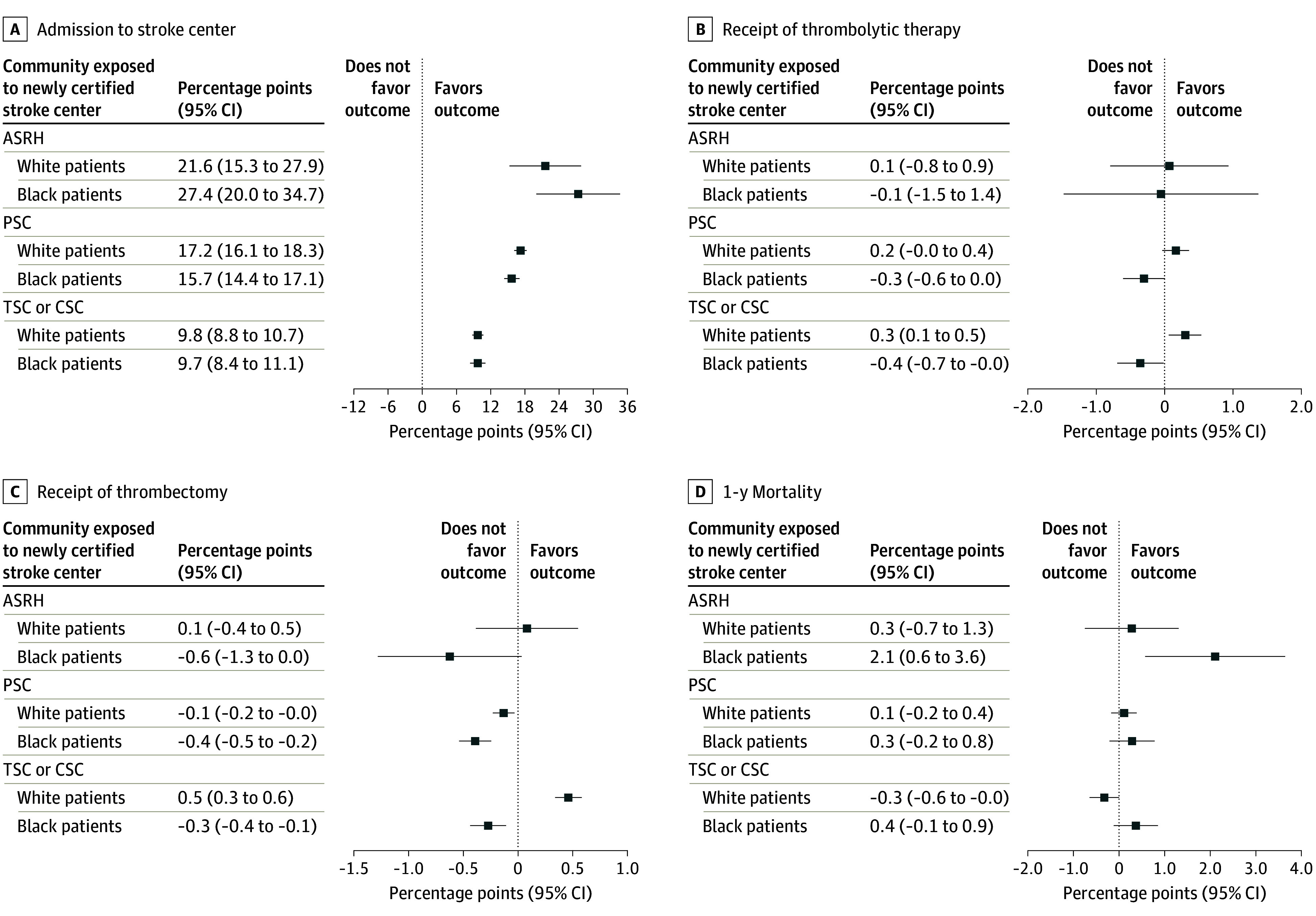
Changes in Probability of Outcomes After Patient Experiences a Newly Certified Stroke Center Within a 30-Minute Drive Time, by Highest Level of Certification Error bars indicate 95% CIs. ASRH indicates Acute Stroke Ready Hospital; CSC, Comprehensive Stroke Center; PSC, Primary Stroke Center; and TSC, Thrombectomy-Capable Stroke Center.

The overall stroke center certification indicator in model 1 could mask differences across certification levels because each certification level has different capacities. Figure 3 shows the results from model 2, for which we separated exposure to various levels (complete results in eTable 2 in Supplement 1). Figure 3A illustrates that stroke center admission was increased most after ASRH certification and least after TSC or CSC certification, likely because many TSC or CSC communities already had stroke services or had upgraded from PSCs. At each level, changes in admission were similar for Black and White patients.

There was no statistically significant change in receipt of thrombolytic therapy when the exposure was to an ASRH or PSC (Figure 3B). However, exposure to a new TSC or CSC was associated with a 0.5-pp increase (95% CI, 0.3-0.6 pp) in thrombectomy for White patients (from a baseline of 1%) and a 0.3-pp decrease (95% CI, −0.4 to −0.1 pp) for Black patients (from a baseline of 1%) (Figure 3C). There was no statistically significant change in being home at 90 days after exposure at all stroke center certification levels. Black patients exposed to a new ASRH had a 1-year mortality rate that was 2.1 pp higher (95% CI, 0.6-3.6 pp) than Black patients in communities without new certification (Figure 3D). Mortality was similar for White patients in communities exposed to new ASRHs. For White patients, mortality rates for those exposed to a TSC or CSC within 30 minutes were 0.3 pp higher (95% CI, −0.7 to −0.0 pp) from a baseline of 32%, while there was no difference for Black patients.

In our sensitivity analysis, we repeated models 1 and 2 with a reduced exposure threshold of 15-minute drive time. Results were qualitatively similar, but effects had a larger magnitude (eFigures 1 and 2 in Supplement 1). For example, exposure to stroke-certified hospitals within 15 minutes was associated with a 0.2-pp increase (95% CI, 0.1-0.3 pp) in thrombectomies for White patients and a 0.2-pp decrease (95% CI, −0.4 to −0.1 pp) for Black patients. White patients also experienced a 0.4-pp reduction (95% CI, −0.7 to −0.1 pp) in 1-year mortality from a baseline rate of 32%, while there was no statistically significant change for Black patients. Our results also remained robust when we included all urban communities regardless of the number of patients in each unit.

## Discussion

This study, analyzing 2.1 million patients with acute stroke across the US from 2009 to 2019, found that while the share of patients receiving thrombolytic therapy and thrombectomy increased over the study period for both Black and White patients, the increase was slower for Black patients compared with White patients. Furthermore, after a stroke center was certified nearby, Black and White patients experienced different, and sometimes opposite, changes. Although we observed similar increases among both groups in admission to certified stroke centers after a nearby opening, changes in the percentage of patients receiving acute stroke treatment differed. Among White patients, exposure to a newly certified stroke center was associated with higher rates of thrombolytic therapy compared with White patients in the control community. For Black patients, however, the likelihood of receiving thrombolysis and thrombectomy decreased after the community experienced newly certified stroke centers relative to Black patients in communities without stroke center expansion. These disparities appeared to be associated with exposure to higher-level stroke centers. White patients had slightly lower 1-year mortality rates and a higher probability of thrombectomy after a newly certified TSC or CSC opened near their community, while the probability of receiving thrombectomy after a TSC or CSC opened nearby was lower among Black patients.

These findings should be interpreted with care. Our findings do not suggest that stroke center certification is, for example, associated with an absolute decrease in the likelihood of thrombolytics or thrombectomy for Black patients, but rather that the increase is smaller than among their Black counterparts in reference communities that did not experience a stroke center opening. In this case, we can see that the disparity between White and Black patients has widened over time with exposure to certified stroke centers, as reflected in Figure 1C.

Our study adds to the current literature by going beyond examining cross-sectional differences over time; rather, we took advantage of the natural experiment of stroke certification rollout over the past decade to capture any differential benefit between Black and White patients with stroke when both were exposed to newly certified centers. Unlike prior studies, we used a community fixed-effects model to account for patient- and community-level differences. This model allows us to isolate changes not associated with baseline clinical or socioeconomic disparities. Our findings suggest that both cross-hospital (ie, White and Black patients present to different stroke hospitals) and within-hospital factors may contribute to the muted associations observed for Black patients. For example, hospitals that historically treat a large share of Black patients may be more crowded or underresourced even after certification.^25,26,27^ Stroke center openings could be a response to the community’s higher need for stroke care (ie, higher disease burden); alternatively, newly certified centers might also be associated with an increased number of stroke diagnoses. Although our analysis cannot identify the precise pathways resulting in the treatment disparity, our results showed that certification efforts over the past decade did not bridge these gaps and were, in fact, associated with exacerbations in the disparity.

We observed that mortality was slightly higher among Black patients in communities with new ASRH certifications compared with those without stroke center upgrades. This finding is difficult to understand, although it should be seen in the context of the data, where very few communities have ASRH openings (only 2% of patients are in such communities), given that ASRHs tend to be in areas with less geographic access to care in general. For Black patients, more than 50% of these ASRH exposures occurred in Georgia, Louisiana, and Texas—states that may differ in unmeasured ways. Despite controlling for secular trends, heterogeneity in how stroke certification affects ambulance routing and transfer protocols across health care markets could explain these disparities.

### Limitations

This study has several limitations. First, Medicare data include only fee-for-service beneficiaries, excluding those enrolled in Medicare Advantage or those younger than 65 years with end-stage kidney disease. Although patients enrolled in Medicare Advantage may be healthier, we do not expect this to affect our findings as we focus on differences between Black and White patients with stroke. Second, there is no central repository for stroke certification data. We obtained data from national and state certification organizations, likely producing the most comprehensive dataset available, although some hospitals may be missing.^11^ This would bias results only if the missing data pattern systematically differed by geography, which we do not expect. Third, drive-time estimates are subject to measurement error due to variability in traffic, time of day, and method of presentation (eg, ambulances with lights and sirens vs private cars). Such errors can lead to attenuation bias, which makes our estimates conservative. Fourth, we were limited to 2 patient outcomes due to data availability: home at 90 days and mortality. Because acute stroke treatments have primarily been shown to be associated with improved functional outcomes rather than mortality, our findings are conservative. Fifth, because community fixed effects are critical to our identification strategy, we chose the LPM over other estimation methods. One problem with our chosen model is out-of-bound estimates. In our analysis, such occurrences were small (between 0% and 3%, depending on the outcome), except for thrombectomy (20% due to this being a very low-probability outcome). Despite this limitation, the LPM remained the best option because it provided consistent and proper estimates in our context, and we did not use estimates. Sixth, fixed-effects models can account for time-invariant unobserved differences across communities but not time-varying ones. Our findings remained robust when we allowed for separate time trends for treatment and control communities in a sensitivity analysis. Seventh, as with all observational studies, residual confounding is possible. Future research with richer clinical data and earlier dates to establish preexposure outcome trends would help validate and extend our findings.

## Conclusions

This cohort study found that, although a newly certified stroke center near a community was associated with similar increases in the likelihood of admission to a stroke center among Black and White patients, there was an increase in stroke thrombolysis rates only for White patients. Thrombectomy-Capable Stroke Center and CSC certification was associated with an increased likelihood of receiving thrombectomy and improved 1-year mortality rates for White patients compared with their White counterparts without newly certified centers nearby. However, Black patients experiencing newly certified TSCs and CSCs near their communities experienced a decreased likelihood of receiving thrombectomy compared with Black patients who did not experience any stroke center openings and had no differential change in mortality. If the benefits associated with stroke center certification are concentrated among White patients and serve to widen rather than narrow the known disparities in acute stroke care, a more targeted approach may be necessary to address stroke care disparities directly.

## Notes

Supplement 1. **eTable 1.** Complete Results of Model 1 **eTable 2.** Complete Results of Model 2 **eFigure 1.** Changes in Probability of Outcomes After Patient Experiences a Newly Certified Stroke Center Within a 15-Minute Drive Time **eFigure 2.** Changes in Probability of Outcomes After Patient Experiences a Newly Certified Stroke Center Within a 15-Minute Drive Time, by Highest Level of Certification

Supplement 2.
Data Sharing Statement
