# Supplementary material for: Outcomes for Black and White Patients After Certification of Nearby Stroke Centers
Source: JAMA Netw Open. 2025 Jul 28;8(7):e2522019. doi: 10.1001/jamanetworkopen.2025.22019 (PMC12305387; doi:10.1001/jamanetworkopen.2025.22019)
Supplement: Supplement 1. — eTable 1. Complete Results of Model 1 eTable 2. Complete Results of Model 2 eFigure 1. Changes in Probability of Outcomes After Patient Experiences a Newly Certified Stroke Center Within a 15-Minute Drive Time eFigure 2. Changes in Probability of Outcomes After Patient Experiences a Newly Certified Stroke Center Within a 15-Minute Drive Time, by Highest Level of Certification [file jamanetwopen-e2522019-s001.pdf]

Supplementary Online Content

Shen YC, Kim AS, Hsia RY. Outcomes for Black and White patients after certification of nearby stroke centers. *JAMA Netw Open.* 2025;8(7):e2522019. doi:10.1001/jamanetworkopen.2025.22019

eTable 1. Complete Results of Model 1

eTable 2. Complete Results of Model 2

eFigure 1. Changes in Probability of Outcomes After Patient Experiences a Newly Certified Stroke Center Within a 15-Minute Drive Time

eFigure 2. Changes in Probability of Outcomes After Patient Experiences a Newly Certified Stroke Center Within a 15-Minute Drive Time, by Highest Level of Certification

This supplementary material has been provided by the authors to give readers additional information about their work.

eTable 1. Complete Results of Model 1

	Admitted to any stroke center	Received thrombolytic therapy	Received thrombectomy	Home at 90 days	1-year mortality
Outcome mean at baseline	58.42%	5.09%	0.72%	64.99%	31.52%
**On and after year-quarter gaining certified stroke center (of any level) within 30 minutes**					
White patients	15.59**	0.20*	0.05	0.08	-0.02
	[14.56,16.62]	[0.02,0.39]	[-0.05,0.14]	[-0.19,0.35]	[-0.28,0.25]
Black patients	14.56**	-0.36*	-0.48**	-0.03	0.48*
	[13.23,15.90]	[-0.65,-0.08]	[-0.61,-0.34]	[-0.49,0.43]	[0.05,0.91]
**Patient characteristics**					
Black	0.20	-1.78**	-0.31**	0.16	-2.29**
	[-0.61,1.01]	[-2.00,-1.55]	[-0.41,-0.20]	[-0.24,0.57]	[-2.66,-1.92]
Female	-0.28**	-0.39**	0.17**	-1.82**	-0.24**
	[-0.38,-0.18]	[-0.48,-0.29]	[0.12,0.22]	[-1.96,-1.68]	[-0.37,-0.10]
Age at time of admission	-0.04**	-0.10**	-0.05**	-1.01**	1.22**
	[-0.04,-0.03]	[-0.11,-0.09]	[-0.05,-0.05]	[-1.02,-1.00]	[1.21,1.23]
Age squared	-0.00**	-0.00**	-0.00**	-0.04**	0.06**
	[-0.00,-0.00]	[-0.00,-0.00]	[-0.00,-0.00]	[-0.04,-0.04]	[0.05,0.06]
**Clinical characteristics**					
Recurrent stroke	-0.05	-2.27**	-0.78**	-4.71**	5.17**
	[-0.20,0.10]	[-2.42,-2.12]	[-0.86,-0.70]	[-4.95,-4.48]	[4.93,5.40]
Peripheral vascular disease	0.65**	-0.55**	0.37**	-0.53**	1.98**
	[0.46,0.84]	[-0.69,-0.40]	[0.28,0.45]	[-0.75,-0.32]	[1.76,2.19]
Pulmonary circulation disorders	0.92**	1.20**	1.07**	-6.46**	8.09**
	[0.65,1.19]	[0.96,1.45]	[0.92,1.21]	[-6.83,-6.09]	[7.71,8.46]
Diabetes	-0.27**	-1.72**	-0.66**	-2.45**	2.30**
	[-0.38,-0.17]	[-1.81,-1.62]	[-0.71,-0.61]	[-2.59,-2.30]	[2.16,2.44]

© 2025 Shen YC et al. *JAMA Network Open.*

Kidney failure	-0.12	-1.41**	-0.82**	-4.81**	7.97**
	[-0.26,0.01]	[-1.52,-1.29]	[-0.88,-0.76]	[-4.98,-4.63]	[7.80,8.15]
Liver disease	0.17	-2.56**	-0.80**	-4.14**	6.62**
	[-0.28,0.62]	[-2.97,-2.15]	[-1.05,-0.55]	[-4.79,-3.48]	[5.97,7.28]
Cancer	0.20	-3.21**	-0.34**	-19.52**	31.12**
	[-0.03,0.43]	[-3.40,-3.01]	[-0.46,-0.21]	[-19.88,-19.16]	[30.76,31.49]
Dementia	-1.34**	-3.69**	-1.97**	-10.59**	11.42**
	[-1.49,-1.18]	[-3.83,-3.55]	[-2.04,-1.89]	[-10.83,-10.35]	[11.18,11.66]
Valvular disease	0.18*	-0.01	0.02	2.23**	-0.45**
	[0.00,0.36]	[-0.16,0.14]	[-0.06,0.10]	[2.01,2.45]	[-0.67,-0.23]
Hypertension	0.28**	0.06	-0.30**	5.90**	-6.58**
	[0.13,0.43]	[-0.07,0.18]	[-0.37,-0.23]	[5.71,6.09]	[-6.76,-6.39]
Chronic pulmonary disease	-0.37**	-0.13*	-0.24**	-3.01**	5.21**
	[-0.50,-0.24]	[-0.25,-0.00]	[-0.30,-0.17]	[-3.19,-2.82]	[5.03,5.39]
Rheumatoid arthritis/collagen vascular disease	-0.07	-0.47**	-0.52**	1.02**	0.76**
	[-0.33,0.20]	[-0.73,-0.21]	[-0.66,-0.39]	[0.64,1.40]	[0.38,1.14]
Coagulation deficiency	1.02**	1.18**	2.14**	-6.55**	7.31**
	[0.79,1.25]	[0.93,1.43]	[1.96,2.31]	[-6.91,-6.19]	[6.95,7.67]
Obesity	0.19*	0.42**	0.38**	1.09**	-3.25**
	[0.00,0.38]	[0.25,0.59]	[0.28,0.49]	[0.87,1.31]	[-3.46,-3.04]
Substance use	0.05	-1.35**	-0.51**	-1.54**	-0.89**
	[-0.25,0.35]	[-1.66,-1.04]	[-0.69,-0.32]	[-1.96,-1.11]	[-1.28,-0.50]
Depression	-0.06	-1.08**	-0.48**	-0.34**	-0.66**
	[-0.26,0.13]	[-1.24,-0.92]	[-0.55,-0.41]	[-0.60,-0.09]	[-0.91,-0.41]
Psychosis	-0.32**	-0.58**	-0.96**	-1.82**	0.52**
	[-0.55,-0.09]	[-0.78,-0.37]	[-1.06,-0.86]	[-2.13,-1.50]	[0.23,0.82]
Hypothyroidism	0.06	0.01	-0.04	1.59**	-1.37**
	[-0.06,0.18]	[-0.11,0.13]	[-0.10,0.03]	[1.42,1.76]	[-1.53,-1.20]

© 2025 Shen YC et al. *JAMA Network Open.*

Paralysis and other neurological disorders	2.12**	9.59**	3.19**	-13.98**	10.07**
	[1.98,2.25]	[9.49,9.69]	[3.14,3.25]	[-14.12,-13.85]	[9.94,10.20]
Chronic peptic ulcer disease	0.35	0.12	2.64**	-8.84**	5.08**
	[-0.31,1.01]	[-0.71,0.96]	[1.98,3.30]	[-10.04,-7.63]	[3.92,6.24]
Weight loss	-0.17	-0.71**	0.84**	-21.54**	20.85**
	[-0.43,0.09]	[-0.93,-0.49]	[0.70,0.99]	[-21.89,-21.18]	[20.50,21.20]
Fluid and electrolyte disorders	-0.32**	-0.36**	1.12**	-11.99**	10.03**
	[-0.45,-0.20]	[-0.47,-0.25]	[1.06,1.19]	[-12.17,-11.82]	[9.87,10.20]
Anemia (blood loss and deficiency)	-0.17*	0.92**	1.18**	-4.59**	5.78**
	[-0.32,-0.02]	[0.78,1.06]	[1.09,1.26]	[-4.80,-4.38]	[5.57,5.99]
Post ICD-10 period	1.18**	1.34**	0.14	3.52**	-6.40**
	[0.75,1.61]	[0.86,1.82]	[-0.09,0.36]	[2.89,4.16]	[-7.00,-5.81]
Admitted in 2009	0.00	0.00	0.00	0.00	0.00
	[0.00,0.00]	[0.00,0.00]	[0.00,0.00]	[0.00,0.00]	[0.00,0.00]
Admitted in 2010	-0.11	0.76**	0.19**	1.16**	-0.67**
	[-0.65,0.42]	[0.58,0.93]	[0.11,0.26]	[0.83,1.49]	[-1.00,-0.35]
Admitted in 2011	3.46**	1.67**	0.18**	3.50**	-3.02**
	[2.75,4.17]	[1.47,1.87]	[0.10,0.27]	[3.15,3.85]	[-3.37,-2.68]
Admitted in 2012	6.13**	2.46**	0.41**	4.22**	-2.93**
	[5.33,6.94]	[2.24,2.67]	[0.32,0.51]	[3.85,4.59]	[-3.29,-2.58]
Admitted in 2013	8.67**	3.66**	0.56**	5.05**	-3.85**
	[7.83,9.51]	[3.43,3.89]	[0.46,0.66]	[4.67,5.43]	[-4.22,-3.48]
Admitted in 2014	9.71**	4.37**	0.75**	5.70**	-4.04**
	[8.86,10.55]	[4.14,4.61]	[0.64,0.85]	[5.33,6.08]	[-4.41,-3.67]
Admitted in 2015	11.23**	5.21**	1.64**	6.96**	-5.15**
	[10.36,12.10]	[4.95,5.47]	[1.52,1.77]	[6.56,7.36]	[-5.55,-4.76]

© 2025 Shen YC et al. *JAMA Network Open.*

Admitted in 2016	10.55**	4.93**	2.10**	7.04**	-1.33**
	[9.57,11.53]	[4.40,5.46]	[1.85,2.36]	[6.30,7.79]	[-2.03,-0.63]
Admitted in 2017	11.88**	5.65**	3.10**	7.95**	-1.57**
	[10.90,12.85]	[5.13,6.18]	[2.84,3.36]	[7.20,8.69]	[-2.28,-0.87]
Admitted in 2018	12.92**	6.22**	4.34**	9.00**	-2.40**
	[11.94,13.91]	[5.68,6.75]	[4.07,4.61]	[8.26,9.75]	[-3.11,-1.70]
Admitted in 2019	14.81**	6.27**	5.10**	10.67**	-2.83**
	[13.81,15.81]	[5.74,6.81]	[4.83,5.37]	[9.92,11.41]	[-3.53,-2.12]
constant	62.55**	2.62**	-0.29**	78.79**	20.00**
	[61.92,63.18]	[2.43,2.80]	[-0.38,-0.19]	[78.49,79.09]	[19.71,20.30]
N	1,808,501	1,808,501	1,808,501	1,808,501	1,808,501

© 2025 Shen YC et al. *JAMA Network Open.*

eTable 2. Complete Results of Model 2

	Admitted to any stroke center	Received thrombolytic therapy	Received thrombectomy	Home at 90 days	1-year mortality
Outcome mean at baseline	58.42%	5.09%	0.72%	64.99%	31.52%
On and after year-quarter gaining ASRH within 30 minutes					
White patients	21.58**	0.07	0.08	0.38	0.28
	[15.31,27.86]	[-0.79,0.93]	[-0.38,0.55]	[-0.83,1.59]	[-0.75,1.31]
Black patients	27.36**	-0.05	-0.62	-1.08	2.11**
	[20.01,34.71]	[-1.47,1.37]	[-1.28,0.03]	[-2.96,0.80]	[0.57,3.64]
On and after year-quarter gaining PSC within 30 minutes					
White patients	17.22**	0.16	-0.13**	-0.00	0.11
	[16.14,18.31]	[-0.03,0.36]	[-0.23,-0.03]	[-0.29,0.28]	[-0.17,0.39]
Black patients	15.71**	-0.30	-0.39**	0.24	0.29
	[14.37,17.05]	[-0.61,0.00]	[-0.54,-0.25]	[-0.29,0.77]	[-0.21,0.78]
On and after year-quarter gaining TSC or CSC within 30 minutes					
White patients	9.75**	0.30*	0.46**	0.15	-0.32*
	[8.79,10.71]	[0.06,0.54]	[0.34,0.58]	[-0.18,0.49]	[-0.65,-0.00]
Black patients	9.73**	-0.35*	-0.27**	-0.13	0.37
	[8.39,11.07]	[-0.69,-0.01]	[-0.44,-0.11]	[-0.64,0.38]	[-0.12,0.85]
**Patient characteristics**					
Black	-0.15	-1.78**	-0.29**	0.15	-2.29**
	[-0.91,0.62]	[-2.00,-1.55]	[-0.40,-0.18]	[-0.25,0.56]	[-2.67,-1.92]
Female	-0.28**	-0.39**	0.17**	-1.82**	-0.24**
	[-0.38,-0.18]	[-0.48,-0.29]	[0.12,0.22]	[-1.95,-1.68]	[-0.37,-0.10]
Age at time of admission	-0.04**	-0.10**	-0.05**	-1.01**	1.22**
	[-0.04,-0.03]	[-0.11,-0.09]	[-0.05,-0.05]	[-1.02,-1.00]	[1.21,1.23]

© 2025 Shen YC et al. *JAMA Network Open.*

Age squared	-0.00**	-0.00**	-0.00**	-0.04**	0.06**
	[-0.00,-0.00]	[-0.00,-0.00]	[-0.00,-0.00]	[-0.04,-0.04]	[0.05,0.06]
**Clinical characteristics**					
Recurrent stroke	-0.07	-2.27**	-0.78**	-4.71**	5.17**
	[-0.22,0.08]	[-2.42,-2.12]	[-0.86,-0.70]	[-4.95,-4.47]	[4.93,5.40]
Peripheral vascular disease	0.67**	-0.55**	0.36**	-0.54**	1.98**
	[0.48,0.87]	[-0.69,-0.40]	[0.28,0.45]	[-0.75,-0.32]	[1.76,2.19]
Pulmonary circulation disorders	0.91**	1.20**	1.07**	-6.46**	8.09**
	[0.64,1.18]	[0.96,1.45]	[0.92,1.21]	[-6.83,-6.09]	[7.71,8.46]
Diabetes	-0.26**	-1.72**	-0.66**	-2.45**	2.30**
	[-0.37,-0.16]	[-1.81,-1.62]	[-0.71,-0.61]	[-2.59,-2.30]	[2.16,2.44]
Kidney failure	-0.13	-1.41**	-0.82**	-4.81**	7.97**
	[-0.26,0.01]	[-1.52,-1.29]	[-0.88,-0.76]	[-4.98,-4.63]	[7.80,8.15]
Liver disease	0.21	-2.56**	-0.80**	-4.13**	6.62**
	[-0.25,0.66]	[-2.97,-2.15]	[-1.05,-0.55]	[-4.79,-3.48]	[5.97,7.28]
Cancer	0.22	-3.21**	-0.34**	-19.52**	31.13**
	[-0.01,0.45]	[-3.41,-3.01]	[-0.47,-0.21]	[-19.88,-19.16]	[30.76,31.49]
Dementia	-1.31**	-3.69**	-1.97**	-10.59**	11.42**
	[-1.47,-1.15]	[-3.83,-3.55]	[-2.04,-1.89]	[-10.83,-10.35]	[11.18,11.66]
Valvular disease	0.21*	-0.01	0.02	2.23**	-0.45**
	[0.03,0.39]	[-0.16,0.14]	[-0.06,0.10]	[2.01,2.45]	[-0.67,-0.23]
Hypertension	0.29**	0.05	-0.30**	5.90**	-6.57**
	[0.14,0.44]	[-0.07,0.18]	[-0.37,-0.23]	[5.71,6.08]	[-6.76,-6.39]
Chronic pulmonary disease	-0.37**	-0.13*	-0.24**	-3.01**	5.21**
	[-0.50,-0.24]	[-0.25,-0.00]	[-0.30,-0.17]	[-3.19,-2.82]	[5.03,5.39]
Rheumatoid arthritis/collagen vascular disease	-0.07	-0.47**	-0.52**	1.02**	0.76**
	[-0.34,0.20]	[-0.73,-0.21]	[-0.66,-0.39]	[0.64,1.40]	[0.38,1.14]
Coagulation deficiency	1.05**	1.18**	2.13**	-6.55**	7.31**
	[0.81,1.28]	[0.93,1.43]	[1.96,2.31]	[-6.91,-6.19]	[6.95,7.67]

© 2025 Shen YC et al. *JAMA Network Open.*

Obesity	0.19*	0.42**	0.38**	1.09**	-3.25**
	[0.01,0.38]	[0.25,0.59]	[0.28,0.49]	[0.87,1.31]	[-3.46,-3.04]
Substance use	0.04	-1.35**	-0.51**	-1.53**	-0.89**
	[-0.26,0.33]	[-1.66,-1.04]	[-0.69,-0.32]	[-1.96,-1.11]	[-1.28,-0.50]
Depression	-0.06	-1.08**	-0.48**	-0.34**	-0.66**
	[-0.25,0.14]	[-1.24,-0.92]	[-0.55,-0.41]	[-0.60,-0.08]	[-0.91,-0.41]
Psychosis	-0.32**	-0.58**	-0.96**	-1.82**	0.53**
	[-0.56,-0.09]	[-0.78,-0.38]	[-1.06,-0.86]	[-2.14,-1.51]	[0.23,0.83]
Hypothyroidism	0.06	0.01	-0.04	1.59**	-1.37**
	[-0.06,0.18]	[-0.11,0.13]	[-0.10,0.03]	[1.42,1.76]	[-1.53,-1.20]
Paralysis and other neurological disorders	2.11**	9.59**	3.19**	-13.98**	10.07**
	[1.98,2.25]	[9.49,9.69]	[3.14,3.25]	[-14.12,-13.85]	[9.94,10.20]
Chronic peptic ulcer disease	0.40	0.12	2.63**	-8.84**	5.08**
	[-0.26,1.06]	[-0.71,0.96]	[1.97,3.29]	[-10.05,-7.63]	[3.93,6.24]
Weight loss	-0.17	-0.71**	0.84**	-21.54**	20.85**
	[-0.43,0.09]	[-0.93,-0.49]	[0.70,0.99]	[-21.89,-21.18]	[20.50,21.20]
Fluid and electrolyte disorders	-0.31**	-0.36**	1.12**	-11.99**	10.03**
	[-0.44,-0.19]	[-0.47,-0.25]	[1.06,1.19]	[-12.17,-11.82]	[9.87,10.20]
Anemia (blood loss and deficiency)	-0.16*	0.92**	1.18**	-4.59**	5.78**
	[-0.31,-0.01]	[0.78,1.06]	[1.09,1.26]	[-4.80,-4.38]	[5.57,5.99]
Post ICD-10 period	1.34**	1.34**	0.13	3.52**	-6.40**
	[0.91,1.77]	[0.86,1.82]	[-0.10,0.35]	[2.89,4.16]	[-6.99,-5.80]
Admitted in 2009	0.00	0.00	0.00	0.00	0.00
	[0.00,0.00]	[0.00,0.00]	[0.00,0.00]	[0.00,0.00]	[0.00,0.00]
Admitted in 2010	-0.53	0.76**	0.22**	1.16**	-0.69**
	[-1.06,0.01]	[0.58,0.94]	[0.15,0.29]	[0.83,1.49]	[-1.01,-0.36]

© 2025 Shen YC et al. *JAMA Network Open.*

Admitted in 2011	2.86**	1.68**	0.23**	3.51**	-3.05**
	[2.14,3.58]	[1.48,1.88]	[0.15,0.32]	[3.15,3.86]	[-3.39,-2.70]
Admitted in 2012	6.54**	2.45**	0.40**	4.22**	-2.91**
	[5.76,7.33]	[2.23,2.67]	[0.31,0.49]	[3.85,4.59]	[-3.27,-2.55]
Admitted in 2013	10.23**	3.64**	0.46**	5.04**	-3.78**
	[9.42,11.05]	[3.40,3.87]	[0.36,0.56]	[4.66,5.43]	[-4.16,-3.40]
Admitted in 2014	11.66**	4.35**	0.63**	5.70**	-3.95**
	[10.83,12.49]	[4.11,4.59]	[0.52,0.73]	[5.32,6.08]	[-4.33,-3.58]
Admitted in 2015	13.57**	5.18**	1.49**	6.95**	-5.05**
	[12.71,14.42]	[4.92,5.44]	[1.37,1.62]	[6.54,7.36]	[-5.45,-4.65]
Admitted in 2016	13.04**	4.90**	1.94**	7.03**	-1.21**
	[12.08,14.00]	[4.37,5.43]	[1.69,2.20]	[6.29,7.78]	[-1.92,-0.51]
Admitted in 2017	14.69**	5.62**	2.92**	7.93**	-1.45**
	[13.73,15.66]	[5.09,6.15]	[2.66,3.18]	[7.18,8.68]	[-2.16,-0.73]
Admitted in 2018	15.94**	6.18**	4.15**	8.99**	-2.27**
	[14.97,16.91]	[5.64,6.72]	[3.88,4.42]	[8.24,9.74]	[-2.97,-1.56]
Admitted in 2019	18.10**	6.23**	4.88**	10.65**	-2.67**
	[17.10,19.10]	[5.69,6.77]	[4.61,5.16]	[9.89,11.40]	[-3.39,-1.96]
constant	62.51**	2.62**	-0.28**	78.79**	20.00**
	[61.90,63.13]	[2.44,2.80]	[-0.37,-0.19]	[78.49,79.10]	[19.71,20.29]
N	1,808,501	1,808,501	1,808,501	1,808,501	1,808,501

© 2025 Shen YC et al. *JAMA Network Open.*

eFigure 1. Changes in Probability of Outcomes After Patient Experiences a Newly Certified Stroke Center Within a 15-Minute Drive Time

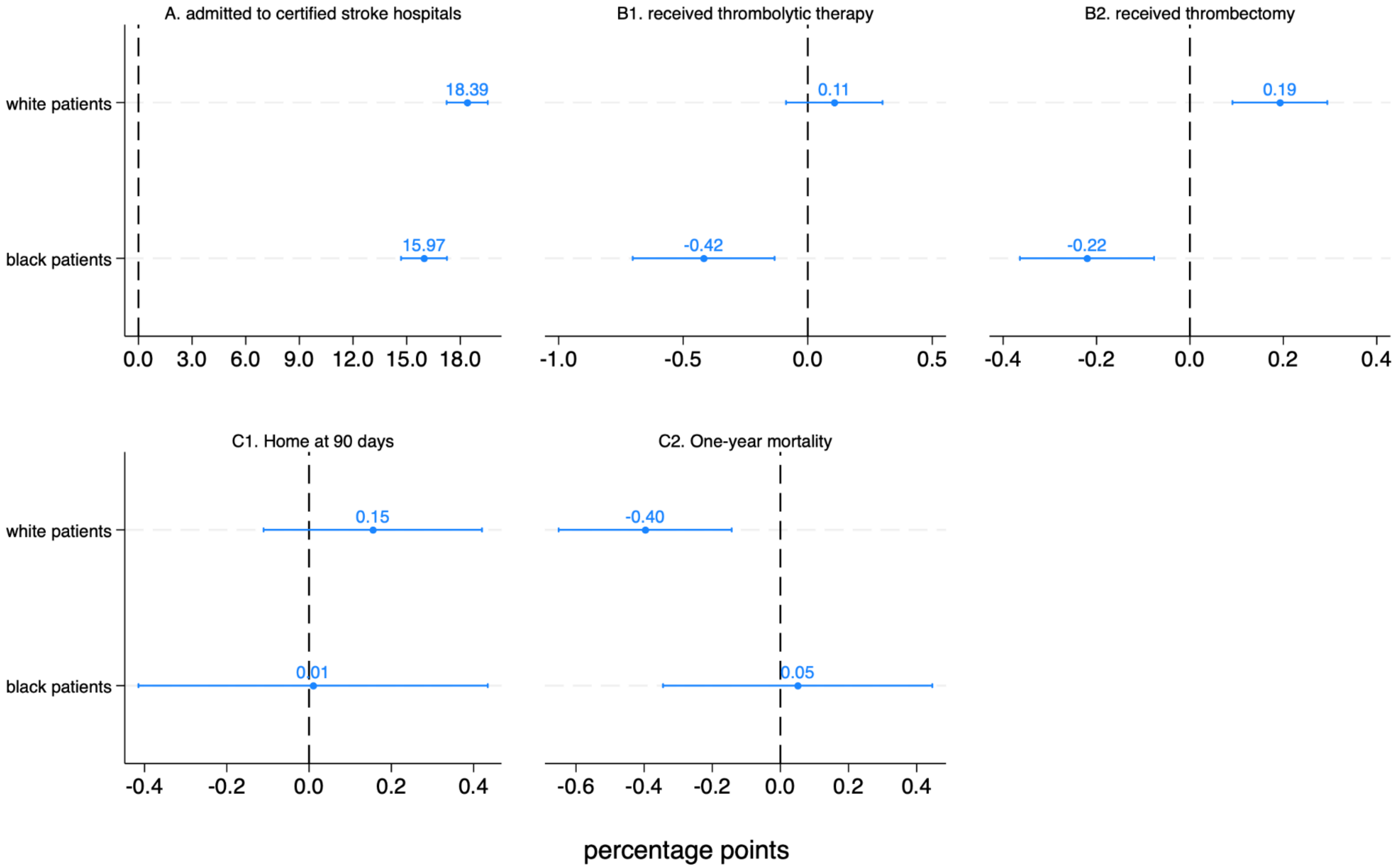

© 2025 Shen YC et al. *JAMA Network Open.*

eFigure 2. Changes in Probability of Outcomes After Patient Experiences a Newly Certified Stroke Center Within a 15-Minute Drive Time, by Highest Level of Certification

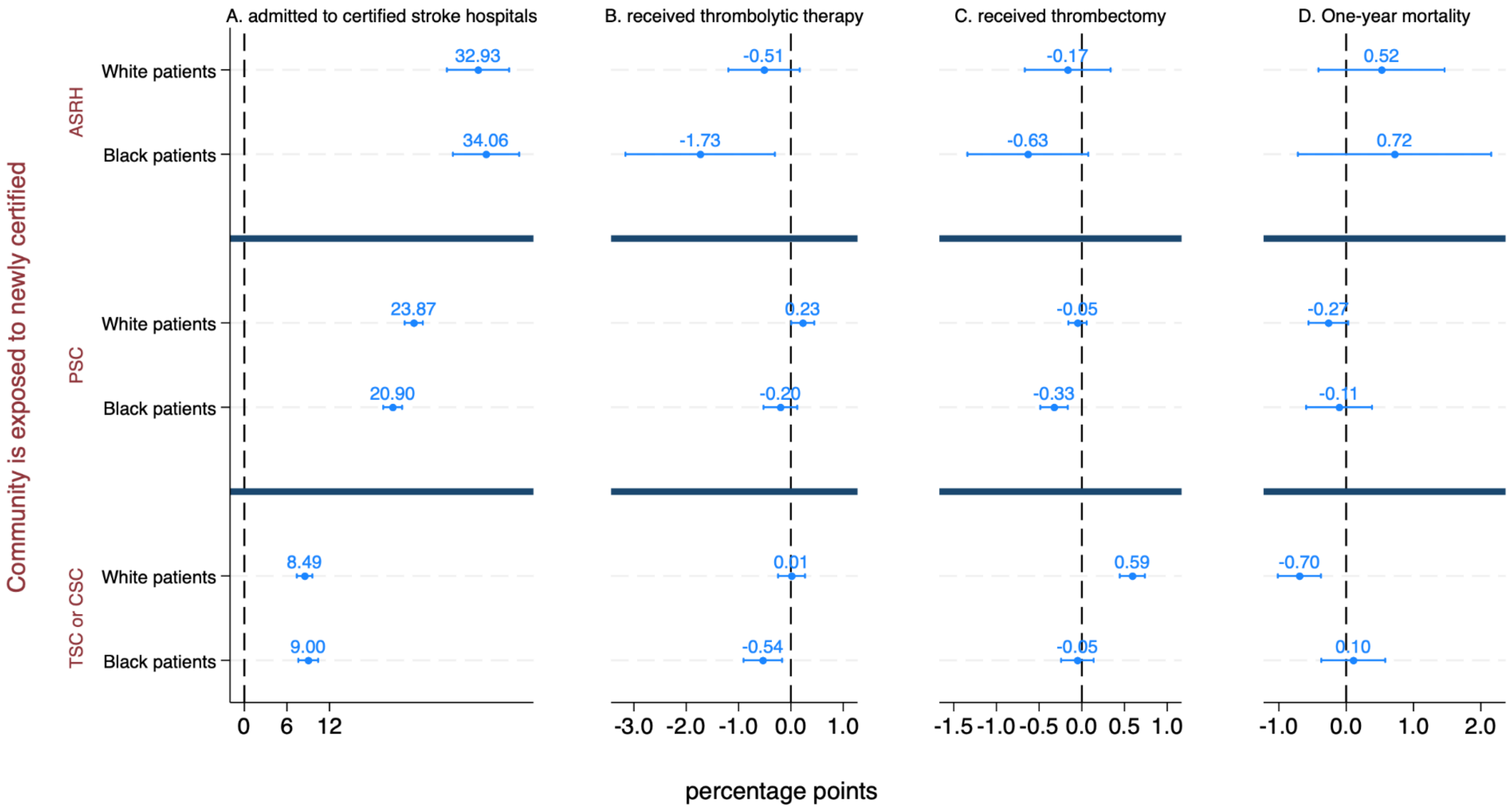

© 2025 Shen YC et al. *JAMA Network Open.*
