# Supplementary material for: Outcomes for Black and White Patients After Certification of Nearby Stroke Centers
Source: JAMA Netw Open. 2025 Jul 28;8(7):e2522019. doi: 10.1001/jamanetworkopen.2025.22019 (PMC12305387; doi:10.1001/jamanetworkopen.2025.22019)
Supplement: Supplement 2. — Data Sharing Statement [file jamanetwopen-e2522019-s002.pdf]

Data Sharing Statement

Shen. Outcomes for Black and White Patients After Certification of Nearby Stroke Centers. *JAMA Netw Open*. Published July 28, 2025. doi:10.1001/jamanetworkopen.2025.22019

Data
Data available: No
